# The Diagnostic Considerations and Clinical Management of Lower Lip Swellings in Adolescents: A Narrative Review

**DOI:** 10.7759/cureus.71707

**Published:** 2024-10-17

**Authors:** Neha Kannan, Karthikeyan Ramalingam, Suvarna Kizhakkoottu, Pratibha Ramani

**Affiliations:** 1 Oral Pathology and Microbiology, Saveetha Dental College and Hospitals, Saveetha Institute of Medical and Technical Sciences, Saveetha University, Chennai, IND

**Keywords:** adolescent, differential diagnosis, fibroma, hemangioma, labial mucosa, lip enlargements, lips, lip swellings, lower lip, mucocele

## Abstract

This article outlines a systematic workflow for the clinical management of lip swellings, ensuring an accurate diagnosis and appropriate treatment plan. The process involves a thorough clinical examination and necessary diagnostic investigations such as imaging and biopsy, which will guide treatment decisions. Depending on the findings, treatment may range from conservative management to surgical excision. Further, we have covered a range of potential conditions including benign lesions such as fibroma, hemangioma, and pyogenic granuloma, as well as malignant neoplasms such as mucoepidermoid carcinoma and adenoid cystic carcinoma. This comprehensive approach will ensure that both common and serious causes of lip swellings are addressed in clinical practice.

## Introduction and background

Lip swellings in the adolescent age group constitute a frequent clinical problem that can result from many causes [1]. These swellings may be congenital, inflammatory, infectious, neoplastic, or traumatic in origin. More common examples of lip swellings include mucocele, fibroma, hemangioma including lobular capillary hemangioma, lymphangioma, lipoma, and malignant tumors. Misdiagnosis or underdiagnosis where the condition may be overlooked altogether is common in the case of lip swellings [2].

This could be attributed to various factors including insufficient knowledge regarding the myriad etiologies of lip swellings, poor physical examination, and the lack of necessary investigations. For this reason, medical personnel need to know the multiple causes of lip swelling in the adolescent age group, as well as how to effectively carry out a physical examination and order appropriate investigations [2-4].

Potential etiologies such as local trauma, allergic reactions, and systemic conditions have also been discussed to provide a comprehensive overview of the approach to diagnosing lip lesions in this age group [5,6]. In this review article, we explain a systematic workflow for the management of different lower lip swellings reported in adolescent patients and all the differential diagnoses to be considered.

## Review

Methodology

The following are the research questions: What are the clinical presentations, histological features, and differential diagnoses of lower lip swellings in adolescents, and how do these factors influence the management and treatment strategies?

A literature search was conducted in the PubMed database to gather relevant studies and articles related to the clinical management and diagnosis of lip swellings in adolescents, covering articles published until 2024. The inclusion criteria for this review focus on studies involving adolescent patients (aged 10-19) presenting with clinical manifestations of lip swellings. These include articles discussing the diagnosis, differential diagnosis, and management of benign and malignant lip swellings. Case reports, case series, systematic reviews, meta-analyses, epidemiological studies involving labial swellings, clinicopathological studies, and review articles published until 2024 were considered along with studies that provide clinical workflows for the diagnosis and treatment of lower lip swellings. The exclusion criteria for this review were studies involving patients outside the adolescent age group (under 10 or over 19). Additionally, articles focusing on systemic and local diseases without any direct presentation of lip swellings, research studies lacking full-text availability, and non-English language studies were excluded.

The following MeSH terms were used: "lower lip" AND "swelling" AND "adolescent," which yielded 24 results. Of these, 15 articles were selected for the initial screening. A manual search identified 30 more articles, 29 of which were screened. After screening all 44 records, 11 were excluded based on the exclusion criteria. Ultimately, 33 articles were selected for review.

Diagnosis of lower lip swellings

**Figure 1:**
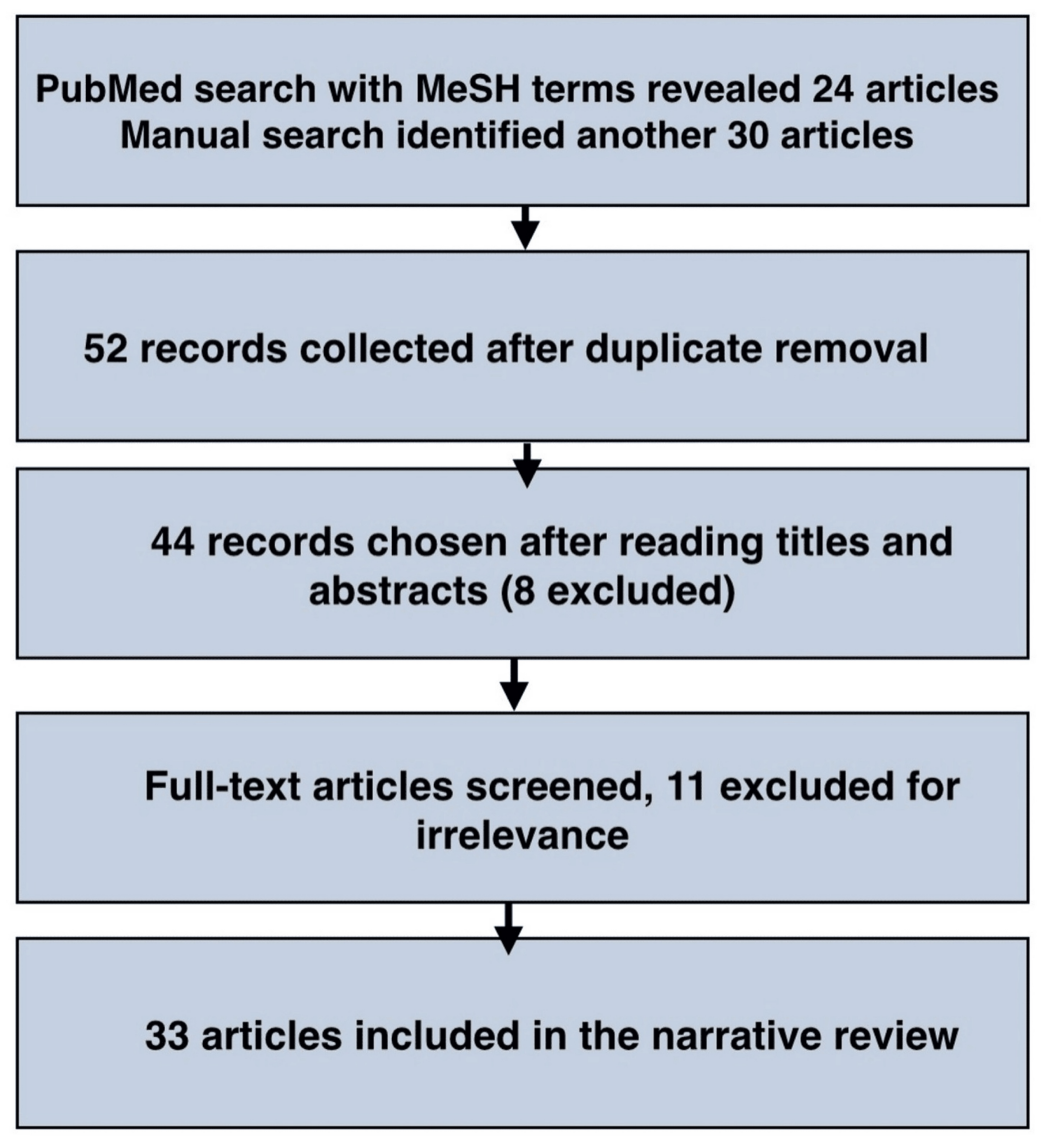
Flow diagram

**Figure 2:**
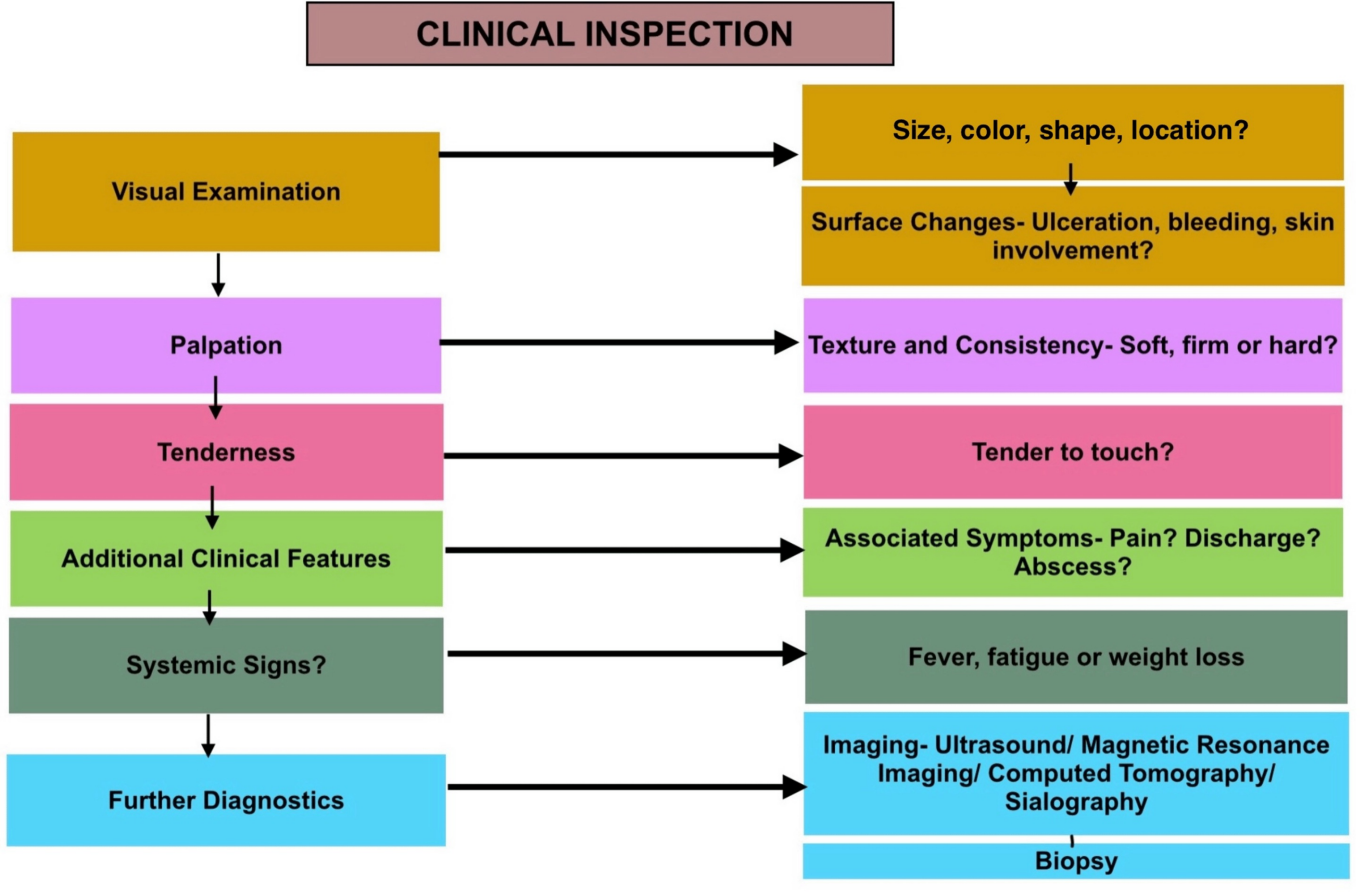
Illustration of the diagnostic workflow for lip swellings Image credits: Neha Kannan and Karthikeyan Ramalingam

Figure 1 shows a workflow that provides a streamlined approach for evaluating patients with lip swellings. It begins with a thorough patient history, including medical, dental, and behavioral factors such as trauma or habits such as lip biting. This is followed by a clinical examination, assessing the swelling's size, location, texture, and duration. Imaging or biopsy may be considered for persistent or suspicious swellings (Figure 2). To manage a patient with lower lip swelling, start by conducting a thorough visual examination of the swelling's size, color, shape, and location [3].

This initial assessment can help identify possible causes such as a mucocele, characterized by a bluish, dome-shaped swelling; a hemangioma, which may appear as a bluish-red swelling that increases with exertion; or an abscess, which is typically erythematous and fluctuant. Examine the surface of the swelling for ulceration, bleeding, or skin involvement. Multiple vesicles that may rupture could indicate herpetic lesions, while a firm swelling with possible ulceration might suggest mucoepidermoid carcinoma. Swollen, everted lips with potential ulcers might point to cheilitis glandularis [4]. Palpate the swelling to assess texture and consistency: soft, compressible swellings could be mucoceles or lipomas, whereas firm swellings might be fibromas, pleomorphic adenomas, or sialolithiasis [5]. Hard swellings could also be granulomas or indicative of conditions such as systemic amyloidosis or sarcoidosis. Evaluate the swelling's tenderness; tenderness might be due to an abscess or trauma from lip biting. Check the swelling's mobility: movable swellings are often lipomas, while fixed lesions might be neoplastic, such as adenoid cystic carcinoma or basal cell carcinoma [5]. Consider associated symptoms such as pain or systemic signs, which could suggest underlying systemic conditions such as systemic lupus erythematosus (SLE) or Behçet's disease [4,5]. Further diagnostics, including imaging or biopsy, may be necessary for persistent or suspicious lesions. Based on the diagnosis, the management plan could involve observation, surgical excision, antibiotic or antiviral treatment, or addressing underlying systemic conditions [6].

**Figure 3:**
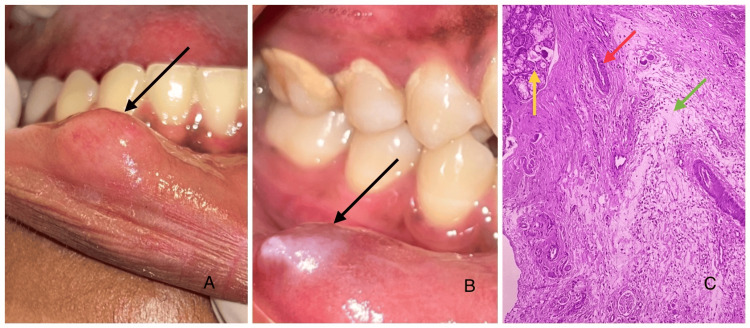
Clinical and microscopic images (A) Solitary, round, soft, dome-shaped swelling (black arrow) on the right labial mucosa with no surface irregularities. (B) Single, ovoid swelling (black arrow) on the right labial mucosa showing a pearly white surface. (C) Photomicrograph of mucocele showing areas of mucin pooling admixed with inflammatory cell infiltrate surrounded by a compressed fibrous connective tissue. Ductal structures and mixed salivary gland acini were also evident. Overlying parakeratinized stratified squamous epithelium, suggestive of present surface epithelium (H&E: 10×). The green arrow points to areas of mucin pooling admixed with inflammatory cell infiltrate; the red arrow points to the ductal structures, and the yellow arrow points to the mixed salivary gland acini. Image credits: authors. Source: department archives, Saveetha Dental College

Lip swellings commonly seen in clinical practice include mucoceles and fibromas. Mucoceles arise from ruptured minor salivary gland ducts. They often affect the lower lip and are enlarged by mucin accumulation (Figure 3) [6].

Fibromas are usually asymptomatic benign nodules caused by chronic irritation or trauma and consist of fibrous tissue proliferation. Other less common swellings, such as pyogenic granulomas and hemangiomas, should also be considered [6,7]. Chaitanya et al. reported that mucoceles were identified as the 17th most common oral lesion [8]. Patil and Maheshwari reported a prevalence of lip lesions in 18.8% of the Indian population, with 29.8% caused by mucoceles, 32.6% by infections, and 20.6% involving premalignant lesions. Males were more affected, with a mean age of 32.6 years [9]. Mathew et al. observed lip lesions, including herpes labialis (0.58%), angular cheilitis (0.58%), and mucoceles (0.16%), in a population from Manipal, Karnataka. Herpes labialis and angular cheilitis were more common in females [10]. Bouquot and Gundlach documented a prevalence of 2.5 cases of herpes labialis and 1.9 cases of angular cheilitis per 1000 individuals in Americans [11].

Detailed differential diagnosis

In Table 1, we have discussed various possible differential diagnoses for lower lip swellings, taking into account both common and rare conditions. This includes entities such as fibromas, hemangiomas, salivary gland tumors, and conditions related to systemic diseases such as sarcoidosis. Proper clinical examination, patient history, and possibly further investigations are essential for arriving at a definitive diagnosis in such cases.

**Table 1:** Differential diagnosis of lower lip swellings This outlines the potential differential diagnoses for lower lip swellings, categorized into congenital, inflammatory, traumatic, infectious, neoplastic, allergic, and systemic causes. It highlights the clinical features and distinguishing characteristics of each condition to aid in accurate diagnosis and appropriate management (adapted and modified from Wong et al., 2020) [6]

Clinical Classification	Lesion Type	Description	Symptoms/Signs
Congenital lesions	Hemangioma	Benign vascular tumor and bluish-red swelling	Red or purple vascular lesion that may grow rapidly in infancy and often involutes by childhood
	Lymphangioma	Soft, compressible swelling, bluish, translucent appearance	Soft, painless, fluid-filled cystic mass, typically present at birth, often associated with lymphatic malformations
	Dermoid cyst	Midline swelling, contains sebaceous material	Firm, non-tender lump containing skin elements and debris, usually located on the midline of the face or neck
	Epidermoid cyst	Slow-growing, firm, round swelling	Round, mobile, and firm cyst under the skin that may contain keratin; can become inflamed or infected
Inflammatory lesions	Mucocele	Soft, bluish, dome-shaped swelling	Swelling of the lip or oral mucosa filled with mucous, often resulting from ductal obstruction; may rupture and heal spontaneously
	Orofacial granulomatosis	Persistent lip swelling	Persistent swelling of the lips and face, often with ulceration, associated with non-caseating granulomas
	Cheilitis glandularis	Inflammation of minor salivary glands	Swelling, tenderness, and crusting of the lip due to the inflammation of salivary glands, often exacerbated by chronic irritation
Infectious lesions	Abscess	Painful, erythematous, fluctuant swelling	Painful, swollen area filled with pus, often red and warm to the touch, accompanied by systemic signs of infection
	Herpetic lesions	Multiple small vesicles that rupture to form ulcers	Painful vesicles or ulcers on the lips or oral mucosa, often recurrent, associated with fever and malaise
	Molluscum contagiosum	Small, umbilicated papules	Small, painless, raised lesions with a central dimple, usually self-limiting and more common in children
Traumatic lesions	Traumatic fibroma	Firm, smooth, painless swelling	Firm, well-defined, painless nodule on the lip or oral mucosa resulting from chronic irritation or trauma
	Lip bite injury	Tender, erythematous swelling	Swelling, bruising, and pain on the lip following self-inflicted trauma, often accompanied by minor bleeding
Neoplastic lesions	Fibroma	Firm, painless mass	Smooth, firm, painless nodules on the lip or oral mucosa, typically slow-growing and benign
	Lipoma	Soft, movable swelling	Soft, mobile, painless subcutaneous mass that feels rubbery, usually slow-growing and benign
	Mucoepidermoid carcinoma	Malignant salivary gland tumor and firm swelling	Swelling or mass in the lip or oral cavity with possible pain and ulceration; may present with regional lymphadenopathy
	Adenoid cystic carcinoma	Malignant tumor, often painful with potential nerve involvement	Slow-growing, painful mass in the salivary glands or oral cavity, often associated with nerve involvement
	Pleomorphic adenoma	Benign mixed tumor, firm and painless mass	Painless, slow-growing, firm mass in the lip or salivary glands; may be mobile and can become malignant if untreated
Allergic/edema-related lesions	Quincke's edema (angioedema)	Acute edema of lower face, eyelids, and lips	Sudden onset of swelling in the lips, the face, and sometimes the airway, often associated with allergies or medications
Systemic disease-related lesions	Hypothyroidism	Chronic lip edema	Symptoms may include dry skin, fatigue, weight gain, and swelling in the face and lips due to myxedema
	Amyloidosis	Chronic, persistent swelling due to abnormal protein deposits	Swelling of the lips and tongue with a waxy appearance, often accompanied by systemic manifestations
	Sarcoidosis	Chronic inflammatory swelling; may cause granulomas	Painless granulomatous lesions on the lips or oral cavity, often with systemic symptoms such as fatigue and respiratory issues
	Melkersson-Rosenthal syndrome	Recurrent or persistent lip swelling, facial paralysis, and fissured tongue	Recurrent swelling of the lips, facial paralysis, and a fissured tongue, with episodes that may resolve spontaneously
	Systemic lupus erythematosus	Painless oral ulcers and erythema, though diffuse swelling may be present	May present with systemic symptoms such as fatigue, joint pain, malar rash, and photosensitivity, which accompany the lip swelling
	Behçet's disease	Painful, recurrent oral ulcers, which can lead to localized swelling of the lower lip	Ulcers frequently appear on the lips and buccal mucosa, and the swelling is often accompanied by other systemic symptoms such as genital ulcers, uveitis, arthritis, and skin lesions

Hemangiomas of the lower lip are benign vascular tumors that present as soft, bluish-purple swellings, often painless, and may fluctuate in size. Hemangiomas are congenital vascular anomalies, where the abnormal proliferation of blood vessels leads to benign tumors, often appearing shortly after birth. Clinically, they are compressible and non-tender and may blanch upon pressure, helping to differentiate them from other lesions. Histopathologically, hemangiomas are characterized by proliferating blood vessels lined by endothelial cells, often with clusters of capillary or cavernous vascular channels depending on the type. They can be identified by their vascular architecture and the lack of cellular atypia [12].

Lymphangiomas, similarly, are congenital malformations of the lymphatic system, where lymphatic vessels fail to connect properly, resulting in fluid-filled masses. Lymphangioma of the lower lip presents as a soft, translucent swelling, often with a bluish or pink hue. Clinically, it is non-tender and poorly defined and may have a "bubble wrap" appearance. Histopathologically, it consists of dilated lymphatic vessels lined by endothelial cells, often infiltrating the surrounding tissues [13].

Dermoid and epidermoid cysts arise from the entrapment of epithelial cells during embryonic development or from trauma. Dermoid cysts in the lower lip are rare, presenting as slow-growing, soft, doughy swellings that are painless and mobile. Clinically, they are well-circumscribed and non-tender. Histologically, they are lined by keratinized stratified squamous epithelium and contain skin appendages such as hair follicles and sebaceous glands [14]. Epidermoid cysts in the lower lip appear as firm, movable, and painless swellings beneath the skin. Clinically, they are smooth, well-circumscribed, and slow-growing. Histopathologically, they are lined by keratinized stratified squamous epithelium, containing keratin debris but no skin appendages [15].

**Figure 4:**
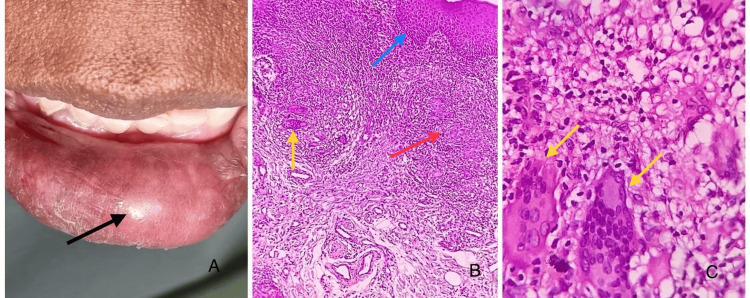
Clinical images and photomicrograph of cheilitis granulomatosis (A) Clinical image showing diffuse swelling involving the lower lip with a glossy surface texture and no surface abnormalities. (B) Photomicrograph showing dense connective tissue stroma with areas of multiple, non-caseating, and well-formed granulomas composed of epitheloid macrophages and multinucleated giant cells (H&E: 10×). The red arrow points to the epitheloid macrophages. The yellow arrow points to the multinucleated giant cells. The blue arrow points to parakeratinized stratified squamous surface epithelium. (C) Photomicrograph showing multinucleated giant cells with eosinophilic cytoplasm and 15-20 hyperchromatic nuclei in the connective tissue stroma (H&E: 40×). The yellow arrows point to the multinucleated giant cells. Image credits: authors. Source: department archives, Saveetha Dental College

Orofacial granulomatosis (Figure 4) is multifactorial, linked to chronic inflammation triggered by infections, food sensitivities, or systemic diseases such as Crohn's. Orofacial granulomatosis presents as persistent, non-tender swelling of the lips, often associated with other facial areas, leading to asymmetry. Clinically, the lips may appear firm and swollen, sometimes with fissuring. Histopathologically, it is characterized by non-caseating granulomas with lymphocytic infiltration and occasional giant cells, typically without necrosis [16].

Cheilitis glandularis involves the inflammation of the minor salivary glands, often due to chronic irritation, sun exposure, or bacterial infection, with smoking and lip trauma as contributing factors, leading to swollen, tender, or ulcerated lips. It manifests as chronic swelling of the lower lip, with eversion and ulceration in advanced stages. Clinically, it presents with multiple enlarged minor salivary glands, and the lip may feel firm or nodular. Histopathologically, it involves dilated and inflamed salivary gland ducts, often with mucous plugging and periductal inflammation, sometimes leading to squamous metaplasia [17].

Abscesses in the lower lip present as painful, swollen, localized collections of pus, often accompanied by redness, warmth, and tenderness. Clinically, they are firm, fluctuating masses that may drain spontaneously. Histopathologically, abscesses consist of a central area of necrotic tissue and pus surrounded by inflammatory cells, predominantly neutrophils [18].

Molluscum contagiosum appears as small, firm, dome-shaped papules with a central dimple, typically painless. The etiology of molluscum contagiosum is viral, caused by a poxvirus, leading to small, painless, dome-shaped lesions on the skin or mucosa. Clinically, they are flesh-colored or pearly and can occur in clusters. Histopathologically, molluscum bodies (large, eosinophilic cytoplasmic inclusions) are seen in the epidermis, with lobular hyperplasia and a central plug of keratin [19].

Herpetic lesions on the lower lip (herpes labialis) present as painful clusters of vesicles that rupture to form shallow ulcers with crusting. They are caused by herpes simplex virus (HSV) infection. Clinically, they are preceded by a tingling or burning sensation. Histopathologically, there is ballooning degeneration of epithelial cells, multinucleated giant cells, and intranuclear inclusion bodies, often seen at the margins of the ulcers [20].

Lip bite injury presents as a painful, swollen area on the lip, often with irregular, torn mucosa and possible bleeding or ulceration. Clinically, it appears as a laceration or bruised area with edema and erythema. Histopathologically, it shows disrupted epithelium with underlying hemorrhage, inflammatory infiltration, and sometimes necrosis or fibrosis during the healing process [21].

**Figure 5:**
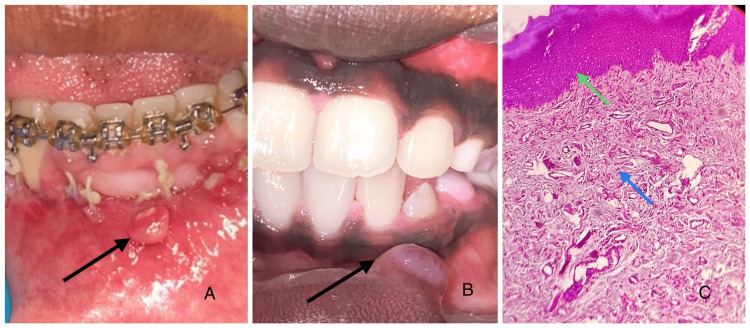
Clinical images and photomicrograph of traumatic fibroma (A) Clinical image showing a round sessile growth on the left labial mucosa with an erythematous surface showing an area of surface ulceration. (B) Clinical image of a round growth on the left labial mucosa with no surface irregularities. (C) Histopathological sections showing haphazardly arranged dense collagen fibers in a fibro-collagenous connective tissue stroma with moderate vascularity and inflammatory cell infiltrate (H&E: 10×). The blue arrow points to the fibro-collagenous stroma. The green arrow points to the hyperkeratinized epithelium. Image credits: authors. Source: department archives, Saveetha Dental College

Traumatic fibroma presents as a firm, painless, smooth, and well-circumscribed nodule on the lip, usually resulting from chronic irritation or trauma. Clinically, it is non-ulcerated and moves freely with the surrounding tissue (Figure 5). Histopathologically, it consists of dense bundles of collagen fibers, surrounded by stratified squamous epithelium, with minimal inflammation [22]. Fibroma of the lower lip presents as a firm, painless nodule that is usually smooth and well-circumscribed. Clinically, it is non-ulcerated and may vary in size, often resulting from chronic irritation or trauma. Histopathologically, it is characterized by dense connective tissue with abundant collagen fibers and minimal inflammatory cells, lined by stratified squamous epithelium [4,22].

Lipoma appears as a soft, movable, and painless mass in the lower lip, often resembling a fatty tissue deposit. Clinically, it is smooth and well-defined and typically presents with a rubbery consistency. Histopathologically, lipomas consist of mature adipocytes within a fibrous stroma, with no signs of inflammation or atypical features [23].

Mucoepidermoid carcinoma of the lower lip typically presents as a painless swelling that may be firm or soft and can vary in size. A common mutation is the *CRTC1*-*MAML2* fusion. Clinically, it may appear as a non-ulcerated lesion that is well-defined but may show infiltrative borders in advanced cases. Histopathologically, it is characterized by a mixture of mucous cells, intermediate cells, and epidermoid (squamous) cells, often arranged in glandular patterns. The presence of varying degrees of keratinization and cellular atypia, along with infiltrative growth patterns, helps distinguish it from benign lesions [24].

Adenoid cystic carcinoma typically presents as a painless swelling in the lower lip, which may be firm and slowly enlarging. They are frequently linked to the *MYB-NFIB* gene fusion. Clinically, it can be mistaken for other benign lesions due to its subtle onset. Histopathologically, it is characterized by cribriform, tubular, or solid patterns, with small nests of epithelial cells surrounded by a myxoid stroma, and may show perineural invasion [25].

Pleomorphic adenoma presents as a painless, well-defined mass, often found in the lower lip, with potential fluctuation in size. The most common genetic alteration involves *PLAG1* gene rearrangements. Clinically, it is firm and mobile, typically without ulceration. Histopathologically, it exhibits a mixture of epithelial and mesenchymal components, with a prominent stroma, and is characterized by diverse cellular morphology [4,26].

The clinical features of Quincke's edema (angioedema) in the lower lip include rapid, painless swelling of the lip, often triggered by allergens, medications, or infections. The swelling is non-pitting and may cause discomfort or difficulty in speaking or eating. Histopathologically, it presents with marked submucosal edema, dilated lymphatic vessels, and mild inflammatory cell infiltration [27]. In hypothyroidism, clinical features may include a swelling in the lower lip due to myxedema. Histopathologically, there is mucin deposition and edema in the connective tissue with sparse inflammatory infiltrates [5,28].

Amyloidosis in the lower lip presents with firm, painless swelling due to amyloid deposition, often accompanied by other systemic signs. Histopathologically, it shows eosinophilic amyloid deposits in the connective tissue, confirmed by Congo red staining. Sarcoidosis in the lower lip is characterized by persistent, painless lip swelling, often associated with other systemic manifestations. Histologically, non-caseating granulomas composed of epithelioid cells, giant cells, and lymphocytes are observed [6,28].

Melkersson-Rosenthal syndrome presents clinically in the lower lip as recurrent, painless swelling, often accompanied by facial palsy and fissured tongue. The lip swelling can be persistent or intermittent, with gradual enlargement over time. Histopathologically, it shows nonspecific chronic inflammation, with granulomatous infiltration, perivascular lymphocytic infiltration, and occasional giant cells. There can also be overlap with findings of sarcoidosis, where non-caseating granulomas may also be present. Both conditions share granulomatous features, but Melkersson-Rosenthal syndrome typically has a more localized presentation with a triad of lip swelling, facial palsy, and tongue fissures [5,6,28].

Lower lip swellings in Behçet's disease and systemic lupus erythematosus (SLE) are both immune-mediated but present differently. In Behçet's disease, the swelling is typically due to painful, recurrent aphthous ulcers on the lips and mucosa, often accompanied by systemic features such as genital ulcers, uveitis, and arthritis. Histopathologically, Behçet's disease shows neutrophilic vasculitis and lymphocytic infiltration. In SLE, lip swelling is less common but may occur alongside painless oral ulcers, erythema, and systemic symptoms such as joint pain and malar rash. Histologically, SLE features immune complex deposition and vasculitis. The etiology of both conditions involves an autoimmune response, with Behçet's disease showing a tendency for recurrent inflammation and SLE involving widespread immune complex formation [28-30].

Cystic swellings can show stratified squamous lining having a granular layer with laminated keratin material [31]. Benign neural tumors such as neurofibroma and schwannoma are the differential diagnosis of lip fibroma. If they undergo cystic change, they may mimic mucocele. Clinically, they present as firm, painful swelling. Histopathologically, these tumors are composed of spindle cells. Schwannoma is composed of Antoni A and Antoni B areas [32,33]. Histopathologically, acute lupus erythematosus shows liquefactive degeneration of the basal layer, upper dermal edema, and scattered interface and perivascular lymphocytic infiltrate, all of which are generally less pronounced as compared to other cutaneous lupus erythematosus subtypes [34,35].

The proper and prompt identification of lip swellings is essential to address any underlying diseases, avoid complications, and warrant timely and proper treatment. The proper documentation of case history is critical for follow-up [30]. Certain swellings, such as mucoceles or fibromas, may be benign and readily treated, while other swellings may be signs of more serious conditions, such as infections, systemic disorders, or malignancies. Early diagnosis can ensure that illnesses that need immediate attention, such as autoimmune disorders or salivary gland tumors, are not missed, lessen patient discomfort, and minimize the chance of complications. The risk of recurrence also decreases when proper identification informs suitable treatment decisions [2,5,6].

Table 2 summarizes the reported cases related to lower lip swellings, including various conditions such as cheilitis, neoplasms, and post-traumatic complications, highlighting key findings and clinical presentations from the literature.

**Table 2:** Various lip swellings in adolescents identified by PubMed search

Author and Year	Reported Case	Case Description
de Sales et al. (2023) [36]	Cheilitis glandularis	A case of cheilitis glandularis with swelling and inflammation of the lower lip, with a systematic literature review
Sofi-Mahmudi (2021) [37]	Oral manifestations in COVID-19 patients	Some patients with COVID-19 may present with lower lip swelling as part of their symptoms
Plaza et al. (2016) [38]	Actinic prurigo cheilitis	Clinicopathological review of 75 cases of actinic prurigo cheilitis affecting the lower lip
Beer et al. (2015) [39]	Lip augmentation complications	Study on hyaluronic acid for lip augmentation; lower lip swelling observed as a side effect
Upadhyay et al. (2017) [40]	Schwannoma	An unusual case of schwannoma presenting as lower lip swelling, diagnosed histologically
Santos et al. (2024) [41]	Foreign body reactions to fillers	Systematic review of foreign body reactions from orofacial fillers, with reports of lower lip swelling
El-Hakim and Chauvin (2004) [16]	Orofacial granulomatosis	A review of six cases of orofacial granulomatosis, including persistent lower lip swelling
Han et al. (2015) [42]	Arteriovenous malformation	A successful treatment case of post-traumatic arteriovenous malformation in the lower lip
Glogau et al. (2012) [43]	Lip augmentation study	A study on the safety of gel particle hyaluronic acid, with few cases showing lower lip swelling
Eivazi et al. (2012) [44]	Port-wine stains	A study showing that port-wine stains on the face may extend to extracutaneous areas such as the lower lip
Ellitsgaard et al. (1993) [45]	Cheilitis granulomatosis in Melkersson-Rosenthal syndrome	Long-term results of surgical cheiloplasty for persistent lower lip swelling in this syndrome
Al-Rawi and Talabani (2008) [46]	Squamous cell carcinoma	A case series analysis of oral squamous cell carcinoma, with some cases involving the lower lip
Zhang et al. (2003) [47]	Traumatic neuroma	A traumatic neuroma in the lower lip arising after laser/cryosurgery for a mucocele
Radhakrishnan et al. (2015) [48]	Embedded tooth fragment	A case of an embedded tooth fragment masquerading as a keloid on the lower lip for 11 months
Rahman et al. (2007) [49]	Extensive hemangioma of the tongue and lip	A case report on external carotid ligation to treat an extensive hemangioma affecting the lower lip

## Conclusions

In conclusion, the early and accurate identification of lip swellings is essential for effective management. This will provide better patient management and outcomes. By recognizing the diverse causes of these swellings, clinicians can provide timely interventions, prevent potential complications, and address any underlying conditions that may otherwise go unnoticed. Following a methodological and strategic workflow will enhance the diagnostic approach in differentiating between inflammatory and neoplastic conditions. Furthermore, a systematic approach fosters better communication among healthcare providers, facilitating collaboration and ensuring that the patients receive a multidisciplinary approach to their treatment. This will ensure that both benign and more serious conditions are appropriately treated, reducing the risk of recurrence and improving overall patient care. Ultimately, this will lead to a more personalized and customized treatment that will benefit the patient.

## Appendices

We have included prevalence studies as they provide valuable population-level data on the occurrence and distribution of lip swellings, particularly in adolescents. These insights help contextualize clinical and histological findings, aiding in identifying patterns that facilitate easier diagnosis and more effective treatment approaches.

Table 3 offers a concise summary of key studies on lower lip swellings in adolescents, which have covered clinical presentations, histological characteristics, and differential diagnoses.

**Table 3:** Summary table

Author	Content	Year	Journal
Mukundan and Ramesh [1]	A case series investigating different treatment approaches for pediatric and adolescent oral mucocele management	2024	Cureus
Bansal et al. [2]	An epidemiological study of 3009 Indian patients presenting with various lip lesions in a tertiary care hospital	2017	Indian Dermatol Online J
Bowers and Schaitkin [3]	Examines management strategies for mucoceles, sialoceles, and ranulas, providing treatment recommendations based on clinical presentations	2021	Otolaryngol Clin North Am
Solanki et al. [4]	Presents two cases to distinguish between traumatic fibroma and mucocele, focusing on clinical features and management	2024	Cureus
Wong et al. [6]	Discusses common causes of swelling in the oral cavity, emphasizing differential diagnoses	2020	Aust J Gen Pract
Sathiyamoorthy et al. [7]	Investigates the prevalence of oral mucocele among outpatients at Saveetha Dental Hospital, providing statistical data	2020	Bioinformation
Chaitanya et al. [8]	A case series on mucocele of the lower lip, detailing clinical presentation, treatment options, and outcomes	2017	Indian Dermatol Online J
Patil and Maheshwari [9]	Study on the prevalence of lip lesions in an Indian population, providing insights into common types and their distribution	2014	J Clin Exp Dent
Mathew et al. [10]	Investigates the prevalence of oral mucosal lesions among patients visiting a dental school in Southern India	2008	Indian J Dent Res
Yanes et al. [12]	Discusses the patterns, outcomes, and implications of hemangiomas of the lip in pediatric and adolescent patients	2016	Pediatr Dermatol
Hasan et al. [13]	Reports a rare case of lymphangioma of the lower lip, discussing diagnostic challenges and treatment options	2022	Case Rep Dent
Rule [14]	A case report on dermoid cyst of the lower lip, emphasizing diagnostic and treatment approaches	1976	Br J Oral Surg
Ito et al. [15]	Reports a rare case of a lip epidermoid cyst caused by a piercing, detailing the clinical management and outcomes	2022	Case Rep Dent
El-Hakim and Chauvin [16]	Reviews six new cases of orofacial granulomatosis presenting as persistent lip swelling, discussing clinical features and management strategies	2004	J Oral Maxillofac Surg
Yanagawa et al. [17]	Two case reports of cheilitis glandularis in Asian-Japanese males, with a literature review of Japanese cases to highlight clinical characteristics	2011	ISRN Dent
Amin et al. [18]	Reviews the diagnosis and treatment of lip infections, providing insights into various infectious etiologies affecting the lip region	2021	J Oral Maxillofac Surg
Svirsky et al. [19]	A report on molluscum contagiosum of the lower lip, discussing presentation and treatment options	1985	Int J Dermatol
de Araújo et al. [20]	A case report on the presentation and treatment of recurrent herpes of the lower lip region using photodynamic therapy and photobiomodulation	2021	Photodiagnosis Photodyn Ther
Limeres et al. [21]	An update on oral self-injury, reviewing various etiological factors, presentations and treatment options for affected patients	2013	Dent Traumatol
Cohen [22]	A case report and literature review on a biting fibroma of the lower lip, occurring at the traumatic site of a tooth bite, detailing management strategies	2022	Cureus
Aita et al. [23]	Reports a case of lipoma on the lower lip, emphasizing diagnostic and therapeutic considerations	2017	J Craniofac Surg
Bhargava et al. [24]	A case report of mucoepidermoid carcinoma of the lower lip, with a review of the relevant literature discussing clinical implications and management	2023	J Oral Maxillofac Pathol
Singer et al. [25]	A case study of adenoid cystic carcinoma located on the lower lip, providing insights into diagnosis and treatment	2021	Dermatol Online J
Nourwali and Dar-Odeh [26]	A case report on pleomorphic adenoma in the lower lip, discussing diagnosis and treatment options, with a review of literature	2019	Eur J Dent
Tackett and Smith [27]	Discusses bupropion-induced angioedema, providing insights into clinical presentation, pharmacological management and patient outcomes	2008	Am J Health Syst Pharm
Gupta et al. [28]	A case report on oral manifestations of hypothyroidism, detailing clinical features and management strategies	2014	J Clin Diagn Res
Błochowiak et al. [29]	Reviews selected presentations of lip enlargement, discussing clinical manifestations and differentiation from other conditions	2018	Postepy Dermatol Alergol
Nigam et al. [31]	A clinicopathological analysis of epidermal cysts, focusing on unusual findings and their clinical significance	2017	Int J Trichology
Haigh et al. [32]	A case report of an unexpected histological diagnosis of a lip schwannoma, discussing diagnostic challenges and treatment approaches	2017	Case Rep Otolaryngol
Nair et al. [33]	Highlights a case of cystic schwannoma that masqueraded as a mucocele, discussing the presentation, diagnostic flow and histological features	2018	J Cytol
